# Hypothesizing that a Pro-Dopaminergic Regulator (KB220z^™^ Liquid Variant) can Induce “Dopamine Homeostasis” and Provide Adjunctive Detoxification Benefits in Opiate/Opioid Dependence

**DOI:** 10.23937/2378-3656/1410125

**Published:** 2016-08-16

**Authors:** Kenneth Blum, Debra Whitney, Lye Fried, Marcelo Febo, Roger L Waite, Eric R Braverman, Kristina Dushaj, Mona Li, John Giordano, Zsolt Demetrovics, Rajendra D Badgaiyan

**Affiliations:** 1 Department of Psychiatry & McKnight Brain Institute, University of Florida College of Medicine, USA; 2 Department of Psychiatry & Behavioral Sciences, Keck School of Medicine of USC, USA; 3 Division of Applied Clinical Research & Education, Dominion Diagnostics, LLC, USA; 4 Division of Neuroscience-Based Therapy, Summit Estate Recovery Center, USA; 5 Division of Clinical Neurology, Path Foundation New York, USA; 6 Division of Personalized Medicine, IGENE, LLC, USA; 7 Division of Molecular Neurobiology, LaVitaRDS, USA; 8 National Institute for Holistic Studies in Addiction, USA; 9 Division of Neuroscience Research and Addiction Therapy, Shores Treatment & Recovery Center, USA; 10 Department of Clinical Psychology and Addiction, Eotvos Lorand University, Hungary; 11 Division of Clinical Addiction Medicine, Pure Recovery, USA; 12 Department of Psychiatry, Laboratory of Molecular and Functional Imaging, University at Minnesota, USA

**Keywords:** KB220Z liquid variant, Opiate/Opioid addiction, Withdrawal, Bup/nx, Detoxification

## Abstract

In order to explore the initiation of detoxification of addictive patients to opiates/opioids (along with some other anti-withdrawal agents), we developed a protocol to be utilized in treatment centers particularly with heavily dependent opiate/opioid subjects. Out of 17 subjects, only three received Buprenorphine/Naloxone (Bup/nx) along with KB220Z. In this pilot, we first used a dose of KB220Z of 2 oz twice daily before meals along with clonidine and benzodiazepines and other anti-nausea and sleep aids including Gabapentin. The dose of KB220Z was maintained for 6 days in five individuals. In a second scenario, we utilized a higher dose of 4 oz every 6 hours, over a 6-day period. The higher dose was employed in another 12 patients.

It is noteworthy that only 3 people have relapsed utilizing these two protocols during the first two weeks of the study, allowing for the remaining 82% to be maintained on KB220Z. The patients have been maintained without any additional Bup/nx for a minimum of 120 days and in one subject, 214 days. We are in the process of testing this hypothesis in multiple treatment centers across the United Sates utilizing data from the Clinical opiate Withdrawal Scale (COWS) pre and post KB220Z. We are in the process of testing this hypothesis in multiple treatment centers across the United Sates.

While this does not constitute an acceptable controlled experiment, it does provide some preliminary evidence that agrees with an earlier study. Moreover, because of the utilization of standard detoxifying agents in this detoxification protocol, we cannot make any inference to KB220Z’s effects. However, out of 17 subjects, only three required Bup/nx suggesting an interesting finding. If further confirmed in larger studies, the utilization for opiate/opioid detoxification may provide a novel way to eliminate the need for addictive opioids during withdrawal and detoxification. This paradigm shift may translate to a reduction in utilizing powerful and addictive opioids like buprenorphine and methadone (especially in these patients at high genetic risk for addiction) as not only detoxifying agents, but also maintenance drugs. While extensive research is required, this pilot paves the way for future investigations that could assist in the reduction of addictive opiate/opioid use and mortalities amongst both the young and old in America.

## Introduction

### Opiate/Opioid epidemic

It is well-established that dopamine signaling is important in the reward pathway and that aberrations in the reward pathway are related to addiction and that by modulating dopaminergic signaling in the reward pathway, we could develop novel treatment modalities for addiction. We are hereby proposing that one way of potentially reducing the out-of control prescribing and subsequent illicit use of opiate/opioids in America is to consider the utilization of Pro-Dopamine Regulation with a nutraceutical complex called KB220Z.

While short-term use of Buprenorphine/Naloxone (Bup/nx) may have some benefit in the maintenance of opiate/opioid dependence, it is still a powerful addictive pharmaceutical that may reduce societal harm on one hand, but promotes addiction on the other hand. There has been a paucity of research related to opiate/opioid dependence withdrawal that evaluates non-addicting non-opiate safe detoxifying agents by promoting “dopamine homeostasis”.

Medication-Assisted Treatment (MAT) is the use of FDA-approved medications, many of which are opioids, for the treatment of opiate/opioid addiction. Two major opioids used to treat opiate/opioid addiction are methadone and buprenorphine, in which the latter has been shown to be nearly 50 times more potent than morphine. Media reports and published drug/addiction policy plans support a greater availability and use of Bup/nx combinations in addiction treatment [1]. Blum et al. have recently commented on raising the limits to 200 patients treated with Bup/nx combinations [2]. Most recently, beginning August 5, 2016, the ceiling will be lifted to 275.

These FDA-approved narcotics in opiate/opioid addiction treatment are definitively habit forming and consequently, addictive just like any other opiate or opioid. They are potentially equally dangerous and capable of the same abuse as any other prescription or illicit narcotic and there is the possibility of street diversion [3].

Previous work from our laboratory found that long-term Bup/nx use lead to blunted emotional responses in its users. Individuals reported less self-awareness of being happy, sad, and anxious. Over time, Bup/nxin effect caused a “zombie-like” effect on its users [4].

Moreover, Methadone was cited in nearly 13% of all the overdose deaths reported in the USA, up from about 4% five years earlier [5]. In 2012, Centers for Disease Control and Prevention (CDC) determined that Methadone was the culprit for one-third of U.S. prescription painkiller deaths. The CDC stated that while Methadone accounts for only 2% of painkiller prescriptions in the U.S., the drug accounts for more than 30% of prescription painkiller overdose deaths. Deaths from Methadone were found to be increasing as prescriptions for the drug rose [6]. The CDC also reported that in 2012, there were 41,502 deaths due to drug poisoning, or drug-overdose deaths, in the U.S.; 16,007 of these deaths involved opioids and 5,925 involved heroin [7].

Statistics provided by the CDC further indicate that 5,282 people died from an accidental overdose of methadone, while 5,925 died from heroin: a difference of 643 people within the same year. Despite empirical data that supports a pattern of increasing deaths attributed to Methadone, and also evidence that demonstrates slim difference between the number of Methadone versus heroin deaths (deaths that Methadone were said to prevent), the FDA-approved MAT narcotic is still widely prescribed, available, and deemed safe by the FDA and other advocates for treating opiate/opioid addicts with opiates/opioids.

However, deaths attributed to Bup/nx are harder to track. According to a *New York Times* report in 2013, the CDC does not track buprenorphine deaths, neither do most emergency rooms, prisons, jails, drug courts, and the majority of medical examiners. A Times analysis of federal data showed that the drug was a “primary suspect” in 420 deaths reported to the FDA since its market availability in 2003. While this may not be medically relevant because of a news report, others have discussed this important issue [8].

To curtail psychoactive drug abuse and dependence, the FDA has approved a number of pharmaceutical agents that either reduce craving or suppress the pleasurable effect of drugs. While such agents have helped many patients, they have not prevented cravings and relapse completely. This fact is underlined by the recent findings that used data from the sophisticated Comprehensive Analysis of Reported Drugs (CARD^™^). The study revealed a significant lack of “compliance” to various treatment medications and lack of “abstinence” from psychoactive drug use in both in-patient and outpatient treatment settings [9].

We agree that short-term use of MAT (possibly from detoxification to less than 12 months), especially Methadone or Bup/nx, may have important benefits regarding preventing unwanted opiate/opioid withdrawal. Moreover, these potent narcotics can induce patient stability ultimately leading to reinstatement of a patient into the workforce and become a so-called “productive member of society,” and thereby, reducing societal harm. Understanding the neuropharmacology of MAT and dopaminergic mechanisms, in which blocking of dopamine induces “psychological extinction” (why use if the thrill is gone), is in our opinion, an inadequate and non-cost-effective way to combat America’s second opiate/opioid epidemic.

### Outlining benefits of buprenorphine and naloxone combinations

Pharmacotherapy in conjunction with controlled withdrawal is currently the most reliable method of opioid detoxification. However, as translational medicine continues to advance and identify genomic markers for opioid sensitivity and dependence, the future holds potential for growth and change [10].

Bup/nx is an effective treatment therapy for opioid dependence and has similar efficacy to Methadone, although more data are needed. Less frequent dispensing of Bup/nx (e.g., thrice weekly) does not appear to compromise efficacy and can improve patient satisfaction. Bup/nx is more effective than clonidine as a medically supervised withdrawal therapy. Moreover, Bup/nx is a well-tolerated medically supervised withdrawal and maintenance treatment. Thus, sublingual Bup/nx is a valuable pharmacotherapy for the treatment of opioid dependence [11].

In another study, Ling et al. [12] compared the effects of a short or long taper schedule after buprenorphine stabilization on patient outcomes, which were measured by opioid-free urine tests at the end of each taper period. They observed that at the end of the taper, 44% of the 7-day taper group (n = 255) provided opioid-free urine specimens, while 30% of the 28-day taper group (n = 261; P = 0.0007) provided opioid-free samples. In addition, there were no reported differences at the 1-month and 3-month follow-ups; the 7-day taper group resulted in 18% and 12% opioid-free specimens, and the 28-day taper group resulted in 18% and 13%, at the 1 month and 3 month follow-ups, respectively). Ultimately, the authors concluded that in individuals terminating buprenorphine pharmacotherapy for opioid dependence, there was no advantage in prolonging the duration of the taper.

While this study provides evidence for the clinical use of Bup/nx in the treatment of opioid withdrawal, as well as favoring short-term use, concerned scientists and clinicians must realize that we are, in essence, only substituting one narcotic for another powerful narcotic. While Bup/nx is a standard in the treatment of opiate/opioid dependence, it is locking people into addiction. Understanding that Bup/nx induces severe withdrawal by itself, and possibly suicide ideation, we attempted to detoxify heavily addicted addicts without Bup/nx.

### Characteristics and neuropharmacology of KB220 variants

With this introduction to the existing problem, we now carried out an experiment utilizing a well-researched amino-acid-enkephalinase inhibition therapy known [13] as Pro-Dopamine Regulator (PDR) for detoxification without the employment of buprenorphine alone or in combination with naloxone in 17 highly addicted opioid addicts. The rationale for the utilization of a KB220 nano-liquid variant is based on a plethora of literature as well as animal and human studies.

Specifically, the patented product is comprised of the following ingredients in validated, evidence-based intake levels: Thiamine, 15 mg (1033% of Daily Value); Vitamin B6, 10 mg (500%); Chromium polynicotinate (as Chrome Mate^®^), 200 mcg (166%); a fixed dose combination of amino acids and herbs called Synaptose^™^, which contains DL-Phenylalanine, L-Tyrosine, and Passion Flower Extract; a Metalloglycoside^™^ Complex containing Arabinogalactans, N-Acetylglucosamine, Astragalus, Aloe Vera, Frankincense Resin, and White Pine Bark Extract; Rhodiola (as RhodiGen^™^); L-Glutamine; 5-Hydroxytryptophan (5-HTP); Thiamine Hydrochloride; Pyroxidal-5-phosphate; and Pyridoxine HCl. The matching placebo powder is also manufactured by Cephram, Inc. (New Jersey). The resultant product utilized is an aqua, nano-liquid product developed in part by Aqua Power (Utah). The rational of the basic ingredients of KB220Z is provided in table 1.

D_2_ receptor activation, especially in the mesolimbic pathway, can be accomplished with “gentle” dopamine (DA) agonist therapy (PDR) involving receptor activation. One possibility is to utilize the nutraceutical complex, KB220Z (PDR), which in pre-clinical and clinical trials, has been shown to mirror the brain reward cascade to potentially induce “dopamine homeostasis.”

In an earlier unpublished, but submitted study, we observed the functional connectivity patterns between several brain structures and areas of the reward system under resting conditions in rats. Experiments were designed to test whether the observed resting state functional connectivity (rsFC) is influenced by administration of a putative dopaminergic agonist, KB220Z^™^ [13].

To understand our rationale for evaluating KB220Z in the present study, we offer the following evidence for clinical benefit of neuromechanisms involved in producing dopamine homeostasis. KB220 variants (nutraceutical complex) have been extensively studied and tested [12]. As reported in detailed review articles [14,15], on both animals and humans to date, many references show that KB220 variants enhance brain enkephalin levels in rodents. Variants of KB220, also have the potential to reduce alcohol-seeking behavior in C57/BL mice and these variants can pharmacokinetically convert ethanol acceptance in preferring mice to non-preferring mice. In humans, KB220Z has demonstrated reduction of drug and alcohol withdrawal symptomatology (i.e., decreased need for benzodiazepines), fewer days with withdrawal tremors, evidence of a lower building up to drink (BUD) score, and elimination of severe depression on the Minnesota Multiphasic Personality Inventory (MMPI). In a double-blind placebo controlled study, patients in recovery treatment had reduced stress response (measured by the skin conductance level [SCL]), and significantly improved Physical Scores and behavioral, emotional, social and spiritual (BESS) scores. There was a six-fold decrease in American Medical Association (AMA) rates when comparing KB220 variants to placebo groups after detoxification. One-year relapse rates, for both cocaine and alcohol abusers, faired significantly better than known average relapse rates for these two psychoactive substances. Healthy volunteers demonstrated an enhanced focus (Table 2).

There is also evidence of reduced craving for alcohol, heroin, cocaine, and nicotine. Also, reductions in inappropriate sexual behavior and reduced post-traumatic stress disorder (PTSD) symptoms, such as lucid nightmares, have been reported [16,17]. Quantitative electroencephalic (qEEG) studies in humans [18] have found that KB220Z modulates theta power in anterior cingulate cortex in abstinent psychostimulant abusers [19]. In abstinent heroin addicts, a single dose of KB220Z compared to placebo in a pilot study [20] resulted in activation of the Nucleus Accumbens (NAc) as well as activation and improvement of the prefrontal-cerebellar-occipital neural network. In addition, it was found that carriers with the DRD2 A1 allele showed a significant Pearson correlation in terms of enhanced compliance to KB220Z treatment relative to carriers of the normal compliment of DRD2 receptors in known obese patients [21]. This further suggests the importance of low dopamine function equating to better treatment outcome. These studies, including double-blinded control studies and others [22–25], have demonstrated positive effects on both craving attenuation and relapse prevention. Most recently, Steinberg et al. [26] showed increased activation of the anterior cingulate brain region using low-resolution brain electromagnetic tomography (LORETA) in attention-deficit/hyperactivity disorder (ADHD) patients.

In fact, it has been shown that DNA-directed compensatory overexpression of the DA D2 receptors (a form of gene therapy) results in a significant reduction in alcohol and cocaine craving behavior in drug-preferring rodents [27–30]. In addition, dopamine D_4_ receptor knockout mice also predict future alcohol intake [31]. Moreover, *in vitro* bromocyrptine induced D_2_ receptor proliferation in rats [32] causes a reduced D_2_ receptor density *in vivo* chronically.

Achieving better treatment outcomes requires an understanding that the maintenance of steady “dopaminergic homeostasis” [33–35] is essential for achieving pleasurable experiences from ordinary daily activities and for relieving stress. Untreated impairments in the homeostatic balance of the DA signaling can facilitate aberrant substance-related disorders and process addictions [36] elucidated in former publications as Reward Deficiency Syndrome (RDS) [37,38]. The scientific research in this field is charged with the challenge of developing non-hazardous, non-addicting agents with dopaminergic up-regulating agonistic properties.

As has been proposed previously, activation, rather than blocking, mesolimbic dopaminergic reward circuitry in the long-term treatment of RDS is the preferred modality even in treating hypersexuality [39,40]. Although, the acute treatment should consist of preferential blocking of postsynaptic NAc DA receptors (D_1_–D_5_), the long-term mesolimbic activation of the dopaminergic system should involve the release and/or activation of DA at the NAc site. There must be focus on balancing the interaction of D_1_ and D_2_ receptor signaling.

This theory suggests that excessive craving behavior can be attributed to reduced number of DA D_2_ receptors, an effect of carrying, for example, the DRD2 A1 allelic genotype; whereas a normal or sufficient density of D_2_ receptors, including other normal genetic expressions such as Orexin and Pro-opiomelacortin (POMC), results in reduced craving and relapse and normal sleep patterns [41,42]. A goal, in terms of preventing substance abuse, could be to induce a proliferation of D_2_ receptors in individuals who are genetically vulnerable. While *in vivo* experiments that use a typical D_2_ receptor agonist induce down-regulation [43], *in vitro* experiments have shown that in spite of genetic antecedents, constant stimulation with a known D_2_ agonist, bromocriptine, results in significant proliferation of D_2_ receptors within the DA system, but chronic treatment of another D_2_ receptor agonist, quinpirole, results in down-regulation, instead of up-regulation or balance proposed for KB220Z and that is a reason for failure in treatment [44].

Based on the current literature, new strategies are needed to treat RDS since the traditionally used therapeutic agents have had limited success in treatment of psychoactive substance abuse and relapse prevention and may require genetic testing leading to personalized medicine [45].

### Rationale

As denoted above, we were compelled to design the current case series and we were optimistic because previous work in our laboratory suggested potential positive clinical outcomes. For example, in 1988, we found improvement in a double-blind evaluation of the nutritional supplement KB220 variant, in a 30-day inpatient alcohol and drug rehabilitation center [22]. The KB220 variant is uniquely designed to elevate levels of enkephalin (s), serotonin, and catecholamines, while balancing GABA, to offset functionally deficient neurotransmitters in alcoholics. Twenty-two patients were studied. The KB220 variant patients, as compared to the control group, (a) had a lower BUD score (1 vs. 2); (b) required no PRN benzodiazepines (0% vs. 94%); (c) ceased tremoring at 72 h, as compared to 96 h; and (d) had no severe depression on the MMPI, in contrast to 24% of the control group. These preliminary data suggest that KB220 variant is a valuable adjunct to therapy by aiding the patient’s physical adjustment to a detoxified state, while facilitating a more positive response to behavioral therapy.

The second article that provided rationale for this experiment involved withdrawal from Bup/nx and maintenance with the putative dopamine agonist KB220 variant. Specifically, it was found that at 432 days post Bup/nx withdrawal, the patient was being maintained on KB220 and had been urine tested and was opioid free [1]. Genotyping data revealed a moderate genetic risk for addiction showing a hypodopaminergic trait [1]. This preliminary case data suggest that the daily use of KB220 could provide a cost-effective alternative substitution adjunctive modality for Bup/nx. The authors encouraged double-blind randomized placebo-controlled studies to test the proposition that KB220 may act as a putative natural opioid substitution maintenance adjunct. More importantly, it is unknown if the KB220 variant could actually be used as a front-line detoxification agent.

### Preliminary evidence of KB220Z liquid as an opiate/opioid detoxification agent

Certainly, there is a great need to find a way to detoxify patients addicted to opiates/opioids, without utilizing either methadone or any other opiate/opioid, including Bup/nx or buprenorphine alone. The main premise here is to detoxify these highly opiate/opioid addicted patients, who are possibly genetically prone to addictive behaviors, by providing endorphinergic-glutaminergic-dopaminergic balance [46] by employing KB220Z liquid variant.

In order to explore the possibility of initiation of detoxification of addictive patients to opiates/opioids (along with some other drugs), we developed a protocol to be utilized in treatment centers with heavily dependent opiate/opioid subjects. The experiment was approved by the PATH Foundation NY IRB committee, and consent forms were filled out by each patient enrolled in the study.

It is to be noted that a treatment center in Los Angeles, CA was selected for this pilot experiment. The center has detoxified many addicted patients and the staff utilizes a number of pharmaceuticals to assist in the detoxification process including: Buprenorphine alone, and in the combined form with naloxone in a 4:1 ratio; Escitalopram: Ondansetron, Gabapentin: Trazodone, Hydroxyzine: Duloxetine, Risperidone: Methocarbamol, Lamotrigine: Quetiapine, Lorazepam: Bupropion, Ketorolac: Clonidine. The doses of these drugs differed across the 17 patients. To determine if KB220z could replace Bup/nx in their regimen, the center carried out detoxification on a number of patients without the administration of Bup/nx by substituting KB220Z.

Out of 17 subjects, because of patient demand, only two received Bup/nx along with KB220Z. In this pilot, we first used a 2 oz dose of KB220Z twice daily before meals along with clonidine and benzodiazepines. The dose of KB220Z was maintained for 6 days in five individuals. In a second scenario, we utilized a higher dose of 4 oz every 6 hours over a 6-day period. It was noted that the intensity of withdrawal symptoms as measured by the Clinical Opiate Withdrawal Scale COWS was reduced with the use of the higher dose. The higher dose was employed in additional12 patients.

It is noteworthy that only 3 subjects have relapsed utilizing these two protocols during the first two weeks of the study, allowing for the remaining 82% to be maintained on KB220Z. The subjects have been maintained without any additional Bup/nx for a minimum of 120 days. However, one patient, a 21-year-old female addicted to methadone and amphetamines, has now been maintained on just KB220Z for about 7 months (Table 3). After 120-day period, the other subjects were no longer followed up, so we are unable to determine how long they have remained on KB220Z. We are in the process of testing this hypothesis in multiple treatment centers across the United Sates.

The following demographics are provided for the initial five patients taking the lower 2 oz dose of KB220Z twice daily, and then maintained on ½ oz of KB220Z twice daily for at least 120 days. The importance here is that during the detoxification period of 6 days, none of these patients required Bup/nx. The center reported that the withdrawal symptomatology was not intense for any of these five patients (Table 3). Unfortunately, these patients have not been followed for the past 120 days, except for the patient cited above. The intensity of withdrawal was assessed by the attending staff and self-reported as well. In a follow-up study we will be able to quantitative the severity of withdrawal symptoms on a larger cohort compared to patients undergoing opiate/opioid withdrawal without KB220Z variant. Until this additional required study is performed we must cautiously interpret this pilot experiment.

As we mentioned the dose for anti-withdrawal pharmaceuticals varied with each patient and to address this issue the following dosage is provided for the five initial participants. Similar dosage was obtained for the additional 12 participants:

#### Patient #1

##### 21-Year-old female

Detoxing from Amphetamines and Meth-Bp 115/60 P 55, Bp 106/75 P 82, Bp 115/78 P 72. Client was taking Synaptamine 2oz once a day, Lamictal 200 mgs once a day, Quetiapine 50 mgs once a day at night for insomnia, Lamotrigine 200 mgs once a day at night for anxiety, Gabapentin 600 mgs four times a day for anxiety, Seroquel 50 mgs at night. Detox was not intense required no Suboxone.

#### Patient #2

##### 32-Year-old male

Detoxing from THC, Benzos, and Oxycontin-Bp 128/88 P 90, BP 137/91 P 90, Bp 145/78 P 86, Bp 149/111 P 84, Bp 129/97 P102, BP 139/84 P85, BP 117/99 P 90, Bp 138/80 P 108, Bp 127/98 83 Bp 153/89 P 83-Client was taking Synaptamine 2 oz two times a day, Lexapro 40 mgs once a day, Zofran as needed for nausea, Gabapentin 600 mgs 1–2 tabs at night as needed for sleep, Trazodone 50 mgs–100 mgs 1–2 tabs at night as needed for sleep, Vistaril 25 mgs – 50 mgs every 4 hours as needed for nausea/vomiting/anxiety. Detox was not intense required no Suboxone.

#### Patient #3

##### 37-Year-old female

Detoxing from Opiod and Meth -BP 139/82 P 83, 78/59 P93, Bp 128/72 P 80, Bp 136/92 88, Bp 113/89 P 79, Bp 120/88 P80, Bp 110/78 P 86-Client was taking Synaptamine 2 oz every morning, Cymbalta 30 mg every morning, Trazadone 25 mgs–50 mgs every night, Clonidine 0.1 mgs once a night, Gabapentin 300 mgs two times a day, Toradol 10 mgs two times a day as needed for pain. Detox was not intense required no Suboxone.

#### Patient #4

##### 30-Year-old male

Detoxing from Heroin and Meth BP 130/90 P 103, Bp 130/83 P 85, Bp 130/83 P 86, Bp 115/80 P 80, Bp 108/66 P 75, Bp 174/95 P 84, Bp 115/90 P 100, Bp 137/83 P 80, Bp 140/102 P 105, Bp 130/85 P 88. Client was taking Synaptamine 2 oz twice a day, Risperdone 1 mg once a day, Robaxin 500 mgs – 1000 mgs four times a day, Gabapentin 300 mgs four times a day, Ativan 0.25 mgs–0.5 mgs every 4 hours, Suboxone 2 mgs/0.5 mgs twice a day for 3 days, Wellbutrin 300 mgs once a day. Detox was not intense required no Suboxone.

#### Patient # 5

##### 34-Year-old male detoxing from meth

Bp 125/88 P 104, Bp 100/70 P 75, Bp 120/70 P 100, Bp 120/80 P 110, Bp 130/60 P 105, Bp 140/104 P 84, Bp 121/77 P 98, Bp 135/102 P 111, Bp 140/98 P 95 Client was taking 2 oz of Synaptamine every morning along with Wellbutrin 150 mgs every morning, Clonidine 0.1 mgs four times a day and Trazodone 50 mgs at night. Detox was not intense required no Suboxone.

Demographics on the additional 12 patients are available from the treatment center and could be retrieved on request by contacting the corresponding author of this article.

### Limits

While this does not constitute an acceptable controlled experiment, it does provide some preliminary evidence that agrees with our earlier case study as previously discussed above, whereby utilizing KB220Z in the nano pill form in a female patient who was maintained opiate/opioid free as measured by a drug-urine screen for at least 432 days [1]. In this earlier study, we could not provide the patient with KB220Z because unlike the liquid form, the patient could not tolerate the pill during intensive withdrawal.

While these results are somewhat encouraging, they do not provide adequate information concerning the real benefit of KB220Z in its liquid form, other than it was safe and easy to take during drug withdrawal when compared to previous tablet forms. Moreover, because of the utilization of a number of standard detoxifying agents utilized in this detoxification protocol, we cannot make any inference to KB220Z’s effects. However, out of 17 patients, only two required Bup/nx suggesting an interesting finding, whereby if further confirmed in larger studies may provide a novel way to eliminate the need for addictive opioids during withdrawal and detoxification followed by maintenance with KB220Z. This must be considered as a pilot and we caution against any interpretation of these preliminary results.

## Discussion

This is the first case report concerning liquid KB220Z and detoxification of known opiate/opioid dependent individuals. In the current study, we decided to evaluate the effect of KB220Z in a nano-liquid with regard to opioid withdrawal symptomatology in 17 patients with a primary diagnosis of drug addiction to opioids or amphetamines/methamphetamine or a combination thereof. The intent of this pilot study was an attempt to withdraw these patients without the use of buprenorphine alone or in combination of naloxone (Bup/nx).

We are cognizant of the important utility of Bup/nx in the short-term treatment of opiate/opioid addiction. It is well known that Bup/nx is a partial μ-opioid agonist combined with the opioid antagonist naloxone in a 4:1 ratio. According to some [47], this combination is safe and beneficial to treat illicit and prescription-opioid dependence. Bup/nx has a lower abuse potential, carries less stigma, and allows for more flexibility than methadone. Bup/nx is indicated for in-patient, ambulatory medically assisted withdrawal (acute detoxification), and long-term substitution treatment (maintenance) of patients who have a mild-to-moderate physical dependence. A stepwise long-term substitution treatment with regular monitoring and follow-up assessment is usually preferred, as it has better outcomes in reducing illicit opioid use, minimizing concomitant risks such as human immunodeficiency virus and hepatitis C transmission, retaining patients in treatment, and improving global functioning. While this seems like a very plausible treatment option approved by the FDA, it is rift with long-term problems as outlined by a number of reports [1,3,15,48–51].

Medically supervised opioid withdrawal is a complex and constantly evolving exercise in multimodal therapy that draws from the expertise of a variety of clinical specialties. In a number of studies to date, it has been reported that acute substitution and weaning off of opioids has been performed utilizing opioid agonists, partial agonists (e.g., buprenorphine), mixed agonist/antagonists (e.g., Bup/nx), and α2 adrenergic agonists with some success.

While thousands of patients are being treated with these ‘classic’ opioid-withdrawal techniques, traditional treatment approaches are being challenged by the emergence of innovative techniques based on an understanding of the neurochemistry of addiction [49,50,52]. Most importantly, Blum’s earlier experiment showing a significant reduction of alcohol withdrawal in a residential United Sates recovery and treatment center has been further validated in this pilot study [22].

## Conclusion

In this pilot observational study, it is noteworthy that only 3 people have relapsed utilizing these two protocols during the first two weeks of the study, allowing for the remaining 82% to be maintained on KB220Z. The patients have been maintained without any additional Bup/nx for a minimum of 120 days and in one subject, 214 days. We are in the process of testing this hypothesis in multiple treatment centers across the United Sates utilizing data from the Clinical opiate Withdrawal Scale (COWS) pre and post KB220Z.

In summary, it is our hypothesis that future steps to overcome the current opiate/opioid epidemic in the United States claiming at least 127 people dying every day from narcotic overdoses must consider newer and better ways to not only detoxify individuals from prescription opioids like methadone and buprenorphine, but induce “dopamine homeostasis” as a maintenance goal free of any opiates/opioids. This is easily said, but may be difficult in practice. In this regard, we encourage additional research in this most perplexing area.

## Figures and Tables

**Table 1:** List of KB220Z Ingredients with targets and mechanism of action.

Ingredient	Therapeutic Target	Mechanism
L-Phenylalanine	Dopamine Synthesis	20% of this precursor amino-acid is converted to dopamine
D-Phenylalanine	Enkephalin/Endorphin Catabolism	Inhibition of the carboxypeptidase (enkephalinase); thereby, increasing opioid peptide levels in brain
L-Tyrosine	Dopamine Synthesis	Rate-limiting step in the synthesis of dopamine
L-Glutamate	GABA Synthesis	Supplied in small amount to assist in balance of over-inhibiting GABA by natural opioid peptides
Chromium Salts	Serotonin Synthesis	Chromium is known to increase the sensitivity of the insulin receptor thereby, reducing the carbohydrate ratio by one-third in the blood; This effect causes gut tryptophan to increase in the brain with a concomitant increase in serotonin synthesis
5-Hydroxytryptophane	Serotonin Synthesis	Involved in the synthetic pathway to produce serotonin
Rhodiola *rosea*	Enzyme Inhibitor Increasing Catecholamines	Rhodiola *rosea* has been shown to inhibit COMT activity thereby, increasing DA in the synapse as well as inhibiting MAO-A in the mitochondria, which increases vesicular DA in pre-synaptic neuron
Pyridoxine Phosphate	Enzyme Catalyst	Assists in the synthesis of dopamine
Passion Flower	Benzodiazepine Receptor Stimulant	By stimulating the benzodiazepine receptor, there is a reduction in anxiety due to stress from detoxification

**Abbreviations:** VTA: Ventral Tegmental Area; NMDA: N-Methyl-D-Aspartate; NAC: Nucleus Accumbens; DA: Dopamine; COMT: Catecholamine-Methyl-Transferase; MAO-A: Monoamine-Oxidase A

**Table 2:** Clinical benefits of KB220Z.

Pre-clinical		
Year	Reference	Key points
1973	Blum K, et al. [53]	Increased brain L-DOPA increases brain dopamine in mice and causes inebriated mice to sleep. Dopamine, 1-tryptophan, and alcohol work similarly in the brain.
1974	Blum K, et al. [54]	When mice were given alcohol and 1-tryptophan or saline, the mice given 1-tryptophan went to sleep. The mice given saline did not. 1-tryptophan and alcohol work similarly in the brain.
1987	Blum K, et al. [55]	Mice genetically predisposed to like alcohol have a measured deficiency in enkephalin. D-phenylalanine and hydrocinnamic acid are substances known to stop the breakdown of enkephalin in the brain-the amount of enkephalin available in the brain increases. When the amount of enkephalin available in the brain increases, both voluntary and forced intake of alcohol decreases. D-phenylalanine is one of the ingredients in NAAT.
Clinical		
Year	Reference	Key points
1988	Blum K, et al. [22]	First small clinical trial of SAAVE (precursor amino acid loading and enkephalinase inhibition-earliest version of NAAT). Designed to elevate levels of enkephalin(s), serotonin, catecholamines, and GABA, thought to be deficient in alcoholics. Compared to controls, those who took SAAVE had lower building up to drink score, required no PRN benzodiazepines, ceased having tremors 24 hours earlier, and had less depression.
	Blum K, et al. [56]	Double-blind placebo-controlled clinical trial of SAAVE of 62 people with Substance Use Disorder (SUD). Results reduced stress as measured by skin conductance, improved Physical and BESS (behavioral, emotional, social and spiritual) Scores, and had a six-fold decrease in leaving Against Medical Advice (AMA) rates.
	Blum K, et al. [57]	Comparison of the effects of Tropamine [T] - (amino acid and vitamin supplement), SAAVE [S]-(a neuronutrient supplement) and no supplement [C] on a group of cocaine abusers in a 30-day hospital treatment program. AMA rate [C] 37.5%, [S] 26.6%, and [T] 4.2%. Tropamine decreased the AMA rate by significant reduction of drug hunger.
1990	Brown RJ, et al. [25]	Relapse prevention using neuro nutrients SAAVE and Tropamine in DUI offenders: either alcohol or cocaine. Reduced relapse rates and enhanced recovery in 10-week outpatient setting. After 10 months’ recovery rate, was SAAVE 73% and Tropamine 53%.
	Blum K, et al. [58]	Examine the effects of PCAL-103 (NAAT) on compulsive eating and weight loss in 27 outpatients attending a supervised diet-controlled treatment program. The PCAL-103 average weight loss was 26.96 lbs vs. 10.2 lbs in the control group. Relapse 18.2% in the PCAL-103 group vs. 81.8% in the control group.
1996	Cold JA, et al. [59]	Small preliminary study of efficacy of NeuRecover-SATM (formerly Tropamine + TM) in the treatment of cocaine withdrawal and craving. Cocaine craving decreased significantly in the Neu Recover-SATM group.
1997	DeFrance JF, et al. [60]	Cognitive processing speeds in normal young adult volunteers were measured before and after 28–30 days of supplementation with a combination of amino acids (NAAT), vitamins, and minerals. Cognitive processing speeds were enhanced by a statistically significant amplitude of the P300 component of the Event Related Potentials (ERPs). Focus improved.
	Blum K, et al. [61]	Of 247 outpatients in a very-low-calorie fasting program, 130 who were having difficulty attaining their desired weight or maintaining their desired weight, constituted the experimental group who took PhenCal^™^ and of the rest, 117 took vitamins and 117 were the control group. The PhenCal^™^ group compared to the control lost twice as much weight, regained 14.7% of the weight, while the control group regained 41.7%, decrease in food cravings for females 70% and males 63%, and decreased in binge eating for females 66% and males 41%.
2001	Ross J, et al. [62]	Preliminary evaluation of six randomly selected former eating disorder female clients (three were also chemically dependent), contacted at 9 months, and 3 years of treatment with amino-acid precursor and enkephalinase inhibition therapy. All 6 reported initial benefit, one relapsed at 6 months, the other 5 all sustained, and in some cases exceeded expectations. 98% of 100 patients similarly treated and evaluated reported significant improvement in both mood and reduced substance craving.
2004	Chen TJ, et al. [63]	A combination of Trexan (a narcotic antagonist) and amino-acids was use to detoxify either methadone or heroin addicts. Results were dramatic in terms of significantly enhancing compliance to continue taking Trexan. Trexan alone for rapid detoxification, the average number of days of compliance calculated on 1000 patients is 37 days. 12 subjects tested, receiving both the Trexan and amino-acid therapy, taking the combination for an average of 262 days. Suggests coupling amino-acid therapy and enkephalinase inhibition, while blocking the delta-receptors with a pure narcotic antagonist as a novel method to induce rapid detox in chronic methadone patients and prevent relapse, and testing this hypothesis with the sublingual combination of the partial opiate mu receptor agonist buprenorphine.
2006	Blum K, et al. [64]	Consumption of large quantities of alcohol or carbohydrates (carbohydrate bingeing) stimulates production and usage of dopamine within the brain. Obesity is due to the need to make up for inadequate dopaminergic activity in the reward center of the brain. This has been called reward deficiency syndrome (RDS) used to categorize such genetic biologic influences on behavior. RDS must be addressed at the same time as behavioral modifications are implemented to adequately treat obese patients. In this small observational trial, 24 individuals completed a survey on which they documented 15 categories of benefit during their experience with a GenoTrim, a NAAT formulation customized to DNA. Statistical analysis of the survey results demonstrated that stress reduction lead to improved sleep, enhanced energy, and improved focus and performance, reduced appetite, loss of unwanted weight, decreased body inches, and enhanced well-being.
2007	Chen TJ, et al. [65]	1-year prospective study that evaluated the effects of taking Haveos (SynaptamineTM) on 61 compliant patients in a comprehensive outpatient clinical program. Results after 12 weeks include significant decrease in craving. Results after 1 year include building up to relapse scores and ability to refrain from drug-seeking behavior both significantly improved. The dropout rate for alcohol users 7% and psychostimulant users 73%.
	Blum K, et al. [66]	In an open clinical study, Amino-Acid Enkephalinase Inhibition Nutraceutical improved symptomatology of 600 recovering Alcoholics. Emotional and behavioral recovery scores significantly improved after administration of oral and intravenous Synaptamine. Mean reductions for craving, depression, anxiety, anger, fatigue, lack of energy and crisis were all significantly greater than 50% (p < 0.001).
	Chen TJH, et al. [67]	Chromium Picolinate (CrP) was tested against placebo in groups of obese patients tested for the Taq1 Dopamine D_2_ Receptor Gene. In carriers of the DRD2 A2 genotype, weight loss and other changes in body composition were significant. They were not significant for patients with the A1/A1 or A1/A2 allele. These results suggest that the dopaminergic system, specifically the density of the D_2_ receptors, confers a significant differential therapeutic effect of CrP in terms of weight loss and change in body fat.
	Blum K, et al. [68]	Preliminary investigational study to evaluate the impact of polymorphisms of five candidate genes on treatment for obesity with NAAT. The formula for each patient was customized based on their genetic results.
2008	Blum K, et al. [69]	A novel experimental DNA-customized nutraceutical, LG839. Polymorphic correlates were obtained for a number of genes (LEP, PPAR-gamma2, MTHFR, 5-HT2A, and DRD2 genes) with positive clinical parameters tested in this study. Significant results were observed for weight loss, sugar craving reduction, appetite suppression, snack reduction, reduction of late night eating, increased energy, etc. Only the DRD2 gene polymorphism (A1 allele) had a significant Pearson correlation with days on treatment.
	Blum K, et al. [21]	Hypothesized that genotyping certain known candidate genes would provide DNA-individualized customized nutraceuticals that may have significant influence on body re-composition by countering various genetic traits. Genotyped for the dopamine D_2_ receptor (DRD2), methylenetetrahydrofolate reductase (MTHFR), serotonin receptor (5-HT2a), Peroxisome Proliferator Activated Receptor gamma (PPAR-γ), and Leptin (OB) genes. Systematically evaluated the impact of polymorphisms of these five candidate genes as important targets for the development of a DNA-customized nutraceutical LG839 [dl-phenylalanine, chromium, l-tyrosine other select amino-acids and adaptogens] to combat obesity with special emphasis on body recomposition as measured by Body Mass Index (BMI). In the 41-day period, we found a trend in weight loss, whereby 71.4% of subjects lost weight.
2009	Blum K, et al. [70]	Brain dopamine has been implicated as the so-called “anti-stress molecule.” The present study investigated anti-anxiety effects of Synaptamine Complex [KB220], a dopaminergic activator, in a randomized double-blind placebo controlled study in alcoholics and in polydrug abusers attending an in-patient chemical dependency program. Patients receiving Synaptamine Complex [KB220] had a significantly reduced stress response as measured by SCL, compared to patients receiving placebo.
2010	Braverman ER, et al. [71]	Case study evaluating sustained weight loss with Synaptamine complex in conjunction with Diethypropion (Tenuate ®), hormonal repletion therapy, use of the Rainbow Diet®, and light exercise. After one year, the 58-year-old patient’s BMI decreased from 32 to 25.4 kg/m^2^ representing a 6.9 kg/m^2^ reduction. His body fat composition decreased from 36.91% to 17.8% as measured by the Hologic DEXA scanner.
	Miller DK, et al. [24]	Intravenous Synaptamine complex in protracted abstinence from alcohol and opiates analyzed by qEEG. Report that the qEEGs of an alcoholic and a heroin abuser with existing abnormalities (i.e., widespread theta and widespread alpha activity, respectively) during protracted abstinence are significantly normalized by the administration of 1 intravenous dose of Synaptamine Complex Variant KB220^™^.
	Blum K, et al. [19]	Protracted Abstinence in Psychostimulant abusers. qEEG analysis in DRD2 A1 allele carriers. Compared to placebo, Synaptose Complex KB220Z^™^ induced positive regulation of the dysregulated electrical activity of the brain in these addicts. 2011
	Blum K, et al. [72]	Synaptamine Complex Variant [KB220]^™^ as an activator of the meso-limbic system and administration significantly reduces or “normalizes” aberrant electrophysiological parameters of the reward circuitry site. Based on our qEEG studies presented herein, we cautiously suggest that long-term activation of dopaminergic receptors (i.e., DRD2 receptors) will result in proliferation of D_2_ receptors leading to enhanced “dopamine sensitivity” and an increased sense of happiness. Oral KB220 showed an increase of Alpha activity and an increase low Beta activity similar to 10–20 sessions with Neurofeedback.
2012	Chen D, et al. [73]	This study examined the effects of combined administration of tyrosine, lecithin, L-glutamine and L-5-hydroxytryptophan (5-HTP) on heroin withdrawal syndromes and mental symptoms in detoxified heroin addicts. The results showed that the insomnia and withdrawal scores were significantly improved over time in participants in the intervention group as compared with those in the control group. A greater reduction in tension-anxiety, depression-dejection, anger-hostility, fatigue-inertia and total mood disturbance, and a greater increase in their vigor-activity symptoms were found at day 6 in the intervention group than in the control placebo group.
	Miller M, et al. [14]	In 129 patients, a combination of IV and oral NAAR therapy (generic KB220) were assessed for Chronic Abstinence Symptom Severity (CASS) Scale over a 30-day period. Three scales were constructed based on this factor analysis: Emotion, Somatic, and Cognitive. All three scales showed significant improvement (P = 0.00001) from pre- to post-treatments: t = 19.1 for Emption, t = 16.1 for Somatic, and t = 14.9 for impaired cognitive. A two-year follow-up in a subset of 23 patients showed: 21(91%) were sober at 6 months with 19(82%) having no relapse; 19 (82%) were sober at one year with 18 (78%) having no relapse; 21(91%) were sober at two-years post-treatment with 16 (70%) having no relapse. Note: these results of cause do not reflect any other recovery skills utilized by the patients including 12 steps program and Fellowship.
	Blum K, et al. [74]	New Definition of Addiction by American Society of Addiction Medicine (ASAM) is based on concepts related to Reward Deficiency Syndrome (RDS). Brain Reward Cascade (BRC) Impairment leads to aberrant craving behavior and other behaviors such as Substance Use Disorder (SUD) due to a “hypodopaminergic” trait/state. Any impairment due to either genetics or environmental influences on this cascade will result in a reduced amount of dopamine release in the brain reward site. After over four decades of development, neuro-nutrient therapy has provided important clinical benefits when appropriately utilized.
2013	Blum K, et al. [1]	A case study of a 35-year-old female in the film industry with a history of chronic pain from reflex sympathetic dystrophy and fibromyalgia. Total monthly prescription costs including supplemental benzodiazepines, hypnotics, and stimulants exceeded $50,000. Withdrawal symptoms were carefully documented when she precipitously stopped taking buprenorphine/naloxone. At 432 days post Suboxone® withdrawal, the patient is being maintained on KB220Z, has been urine tested and is opioid free. Genotyping data revealed a moderate genetic risk for addiction showing a hypodopaminergic trait.
2015	McLaughlin T, et al. [16]	Lucid dreams may be associated with psychiatric conditions, including Post-Traumatic Stress Disorder (PTSD) and Reward Deficiency Syndrome-associated diagnoses. We present two cases of dramatic alleviation of terrifying lucid dreams in patients with PTSD. The medication visit notes reveal changes in the frequency, intensity, and nature of these dreams after the complex putative dopamine agonist KB220Z was added to the first patient’s regimen. The second PTSD patient, who had suffered from lucid nightmares, was administered KB220Z to attenuate methadone withdrawal symptoms and incidentally reported dreams full of happiness and laughter.
	McLaughlin T, et al. [17]	Lucid dreams could be unpleasant or terrifying, at least in the context of patients who also exhibit characteristics of Reward Deficiency Syndrome (RDS) and Post-Traumatic Stress Disorder (PTSD). We present eight clinical cases, with known substance abuse, childhood abuse, and diagnosed PTSD/RDS. The administration of a putative dopamine agonist, KB200Z^™^, was associated with the elimination of unpleasant and/or terrifying, lucid dreams in 87.5% of the cases presented, whereas one very heavy cocaine abuser showed a minimal response. These results required the continuous use of this nutraceutical. If these results, in a small number of patients are indeed confirmed, we may have found a frontline solution to a very perplexing and complicated symptom known as lucid dreams.
	Blum K, et al. [20]	Willuhn et al. reported that cocaine use and even non-substance-related addictive behavior increases as dopaminergic function is reduced. Chronic cocaine exposure has been associated with decreases in D_2_/D_3_ receptors and was also associated with lower activation of cues in occipital cortex and cerebellum, in a recent PET study by Volkow et al. KB220Z induced an increase in BOLD activation in caudate-accumbens-dopaminergic pathways compared to placebo following 1-hour acute administration in abstinent heroin addicts. Increased functional connectivity was observed in a putative network that included the dorsal anterior cingulate, medial frontal gyrus, nucleus accumbens, posterior cingulate, occipital cortical areas, and cerebellum. Results suggest a putative anti-craving/anti-relapse role of KB220Z in addiction by direct or indirect dopaminergic interaction.
2016	McLaughlin T, et al. [75]	The four patients initially reported a gradual but, then, complete amelioration of their long-term, terrifying, lucid dreams, while taking KB220Z. The persistent amelioration of these dreams continued for up to 12 months, after KB220Z. These particular cases raise the scientific possibility that KB200Z increases both dopamine stability as well as functional connectivity between networks of brain reward circuitry in both rodents and humans. In order to attempt to understand the possibility of neuroplasticity, we evaluated the effect of KB220Z in non-opioid-addicted rats utilizing functional Magnetic Resonance Imaging methodology. While we cannot make a definitive claim because rat brain functional connectivity may not be exactly the same as humans, it does provide some interesting clues. We did find following seeding of the dorsal hippocampus, enhanced connectivity volume across several Regions of Interest (ROI), with the exception of the pre-frontal cortex. Interestingly, the latter region is only infrequently activated in lucid human dreaming, when the dreamer reports that he/she had the thought that they were dreaming during the lucid dream.
	Harriet Beitscher-Campbell, et al. [76]	While there are still a number of scientists that would argue the commonality between these two seemingly diverse substances, the field is rift with many neuroscience imaging studies that show a neurochemical commonality as well as other genetic studies showing a hypodopaminergic trait. While we did not provide evidence showing any potential difference among those with anorexia nervosa, binge eating disorder, bulimia nervosa, sub-threshold bingeing, we are reporting at a minimum co–morbidity with eating disorders and SUD. Here we show fifty case reports derived from two independent treatment centers in Florida, that suggest the commonality between food and drug addiction.
	Bruce Steinberg, et al. [26]	Attention Deficit-Hyperactivity Disorder (ADHD) often continues into adulthood. Recent neuroimaging studies found lowered baseline dopamine tone in the brains of affected individuals that may place them at risk for Substance Use Disorder (SUD). This is an observational case study of the potential for novel management of Adult ADHD with a non-addictive glutaminergic-dopaminergic optimization complex KB200Z. Low-resolution electromagnetic tomography (LORETA) was used to evaluate the effects of KB220Z on a 72-year-old male with ADHD, at baseline and one hour following administration. The resultant z-scores, averaged across Eyes Closed, Eyes Open, and Working Memory conditions, increased for each frequency band, in the anterior, dorsal, and posterior cingulate regions, as well as the right dorsolateral prefrontal cortex during Working Memory with KB220Z. These scores are consistent with other human and animal neuroimaging studies that demonstrated increased connectivity volumes in reward circuitry and may offer a new approach to ADHD treatment. However, larger randomized trials to confirm these results are required.

**Table 3:** Demographics of five poly-drug abusing patients detoxified with KB220Z without Bup/nx.

Drug Dependence Type	Gender	Age (Years)	KB220Z detoxify dose 2 oz twice daily with other drugs	Withdrawal Intensity	Minimal Days Maintained on KB220Z at 1/2 oz twice daily
Amphetamines & Methadone	Female	21	Quetiapine Lamotrigine Gabapentin	Not Intense	214
Cannabis, Benzodiazepines & Oxycodone	Male	32	Escitalopram Gabapentin Ondansetron Trazodone Hydroxyzine	Not Intense	120
Methadone & Opioids	Female	37	Duloxetine Gabapentin Trazodone Clonidine	Not Intense	120
Heroin & Methadone	Male	30	Risperidone Methocarbamol Gabapentin Bupropion Lorazepam	Not Intense	120
Methadone	Male	34	Bupropion Trazodone Clonidine	Not Intense	120
